# Octadecyltrichlorosilane (OTS)-coated ionic liquid drops: Micro-reactors for homogenous catalytic reactions at designated interfaces

**DOI:** 10.3762/bjnano.3.4

**Published:** 2012-01-12

**Authors:** Xiaoning Zhang, Yuguang Cai

**Affiliations:** 1 Department of Chemistry, University of Kentucky, 505 Rose Street, Lexington, KY 40506, USA

**Keywords:** AFM, catalyst encapsulation, chemical pattern, ionic liquid, OTS

## Abstract

An ionic liquid (IL), 1-butyl-3-methylimidazolium chloride ([Bmim]Cl) can assemble on prefabricated carboxylic acid–terminated chemical patterns on octadecyltrichlorosilane (OTS) film. The chemical pattern controls the position, shape and size of the IL on the surface. After the IL assembly – by incubating IL drops assembled on sample surface in an OTS silane vapor – an OTS layer was coated on the IL drop surface which encapsulated the IL drop. The OTS-coated capsule can exist stably under aqueous solution. The OTS coating protected the IL drops from being instantaneously dissolved by other solutions. We found that a homogenous catalyst (FeCl_3_) dissolved in [Bmim]Cl can be assembled together on the chemical patterns and subsequently encapsulated together with [Bmim]Cl by OTS coating. The pinhole defects within the vapor-coated silane layer provide space for the catalyst inside the capsule and reactants outside the capsule to meet and react. When the OTS-coated capsule containing a FeCl_3_/IL mixture was soaked under H_2_O_2_ solution, the Fe^3+^ ions catalyzed the decomposition reaction of hydrogen peroxide at the vapor-coated OTS-water interface. Since the shape and position of the interface is defined by the underneath chemical pattern, our findings show that the OTS-coated IL drops assembled on chemical patterns can be used as novel micro-reactors. This allows homogenous catalytic reactions to occur at the designated interfaces.

## Introduction

Ionic liquids (ILs) have promising applications as environmentally friendly solvents [1–2]. Ionic liquids are low temperature melting salts with very low vapor pressure. Thanks to their low vapor pressure, ILs are ideal extraction solvents or reaction media because simple evaporation methods can be used to separate solutes from ILs [3]. In addition, ILs can be custom-made with targeted functions. Because of these advantages, ILs have been engineered as extraction solvents, reaction media and drug delivery materials [4–5]. In most IL applications – such as extraction, lubrication, IL super capacitors – the core function of the IL occurs at the ionic liquid–solid interfaces.

ILs are different from conventional molecular liquids because no individual molecule exists in the liquid. Moreover, ILs are not diluted electrolyte solutions either. Hence, no existing theory and model can precisely describe the behavior of ILs, especially at the IL interfaces. Therefore, studies of the IL interfacial properties are necessary for further developments of IL-based applications. Furthermore, new applications – such as IL reactor, IL-circuit, and surface pattern visualization – require the precise control over the position of the IL drop on surface [6–8].

In this letter, we report studies of the chemical pattern-directed assembly of IL on surface. We found that the chemical patterns can control the shape, size and position of the IL on surface. Furthermore, IL drops on surface can be coated with a layer of silane film, forming an IL capsule. We discovered that the homogenous catalyst FeCl_3_ could be encapsulated together with IL. The pinhole defects on the OTS coating layer provided spaces for the catalyst inside the capsule and reactants outside the capsule to react. Hence, the coated IL drops enable the interfacial chemical reactions.

## Results and Discussion

### Chemical pattern-directed assembly of IL on surface

**Figure 1:**
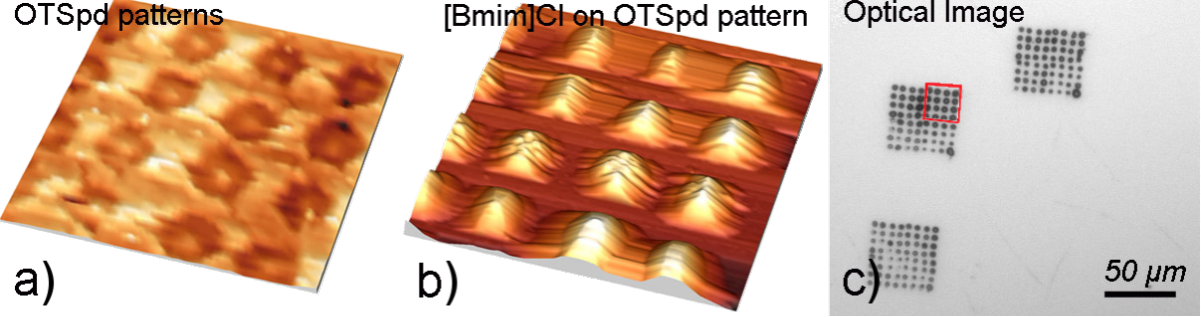
[Bmim]Cl assembles on the OTSpd pattern. a) OTSpd discs fabricated by scanning probe deep oxidation lithography on OTS film. Topography image. The center-to-center distance between two neighboring discs is ~7 μm. b) The same area after [Bmim]Cl was coated on the sample. [Bmim]Cl selectively assembled on the lyophilic OTSpd discs. Topography image. c) Optical image of [Bmim]Cl assembled on the OTSpd patterns. The light background is the OTS film. The red box is the zone shown in a) and b). In this optical image, each IL drop assembled on an OTSpd disc appears as a dark dot.

The carboxylic acid-terminated chemical patterns (partially degraded octadecyltrichlorosilane, OTSpd) were fabricated on the self-assembled monolayer of octadecyltrichlorosilane (OTS) film using the scanning probe deep oxidation lithography method [9]. The OTSpd pattern is a high energy, lyophilic surface whereas the OTS background is a methyl-terminated, low energy, lyophobic surface. Based on the wetting-driven assembly approach [10], liquid can be assembled on the chemical patterns due to the contrast in surface energy [11–12]. Figure 1a shows a representative OTSpd disc array. Figure 1b shows the same region after a liquid [Bmim]Cl drop rolled over the OTSpd discs. By comparing Figure 1a with Figure 1b, we found that the IL micro-drops were selectively deposited on the high-surface energy OTSpd chemical patterns. Figure 1c is the optical image of the IL drop arrays assembled on OTSpd patterns. The background is the OTS film. Each dark spot in Figure 1c is an IL drop assembled on the OTSpd disc. The regions shown in Figure 1a and 1b are highlighted within the red box in Figure 1c.

Figure 1c reveals that the amounts of IL assembled on each OTSpd disc are similar but not identical. Some discs appear darker than the rest, indicating that more IL was assembled on that OTSpd disc. Correspondingly, in the atomic force microscopy (AFM) topography image (Figure 1b), the height of IL drops varies between 250–800 nm. The AFM image reveals more details about the shape of the IL droplet assembled on the OTSpd discs. A representative high-resolution AFM image of the IL drop is shown in Supporting Information File 1, Figure S1. The AFM topography image shows that the IL is not a hemispherical drop that covers the whole OTSpd disc. Instead, the IL adopts a Mexican hat shape – a partial drop sitting on top of a precursor layer. In the OTSpd disc center is the partial drop, which only covers the central part of the OTSpd disc and is typically 250–800 nm high. The central drop is surrounded by a rim, which extends out and covers the remaining part of the OTSpd disc. The rim is thickest at the foot of the central drop and gradually becomes thinner as it extends out. The Mexican hat shape indicates that the IL drop co-exists with an IL precursor layer (the “rim”). The observed Mexican hat shape for an IL drop is not a surprise. Since first discovered by Hardy in 1919 [13], the existence of the precursor layer of a drop on a solid surface has been extensively studied. In fact, the Mexican hat shape has been confirmed as the real shape for most liquid drops on solid surfaces, provided that the drop can be resolved with a sufficient resolution [14].

[Bmim]Cl is miscible with water. When the sample shown in Figure 1c was immersed in water, all IL micro-droplets on the patterned area disappeared instantaneously, indicating that the IL micro-droplets were dissolved in water.

### Silane-coated IL capsules

**Figure 2:**
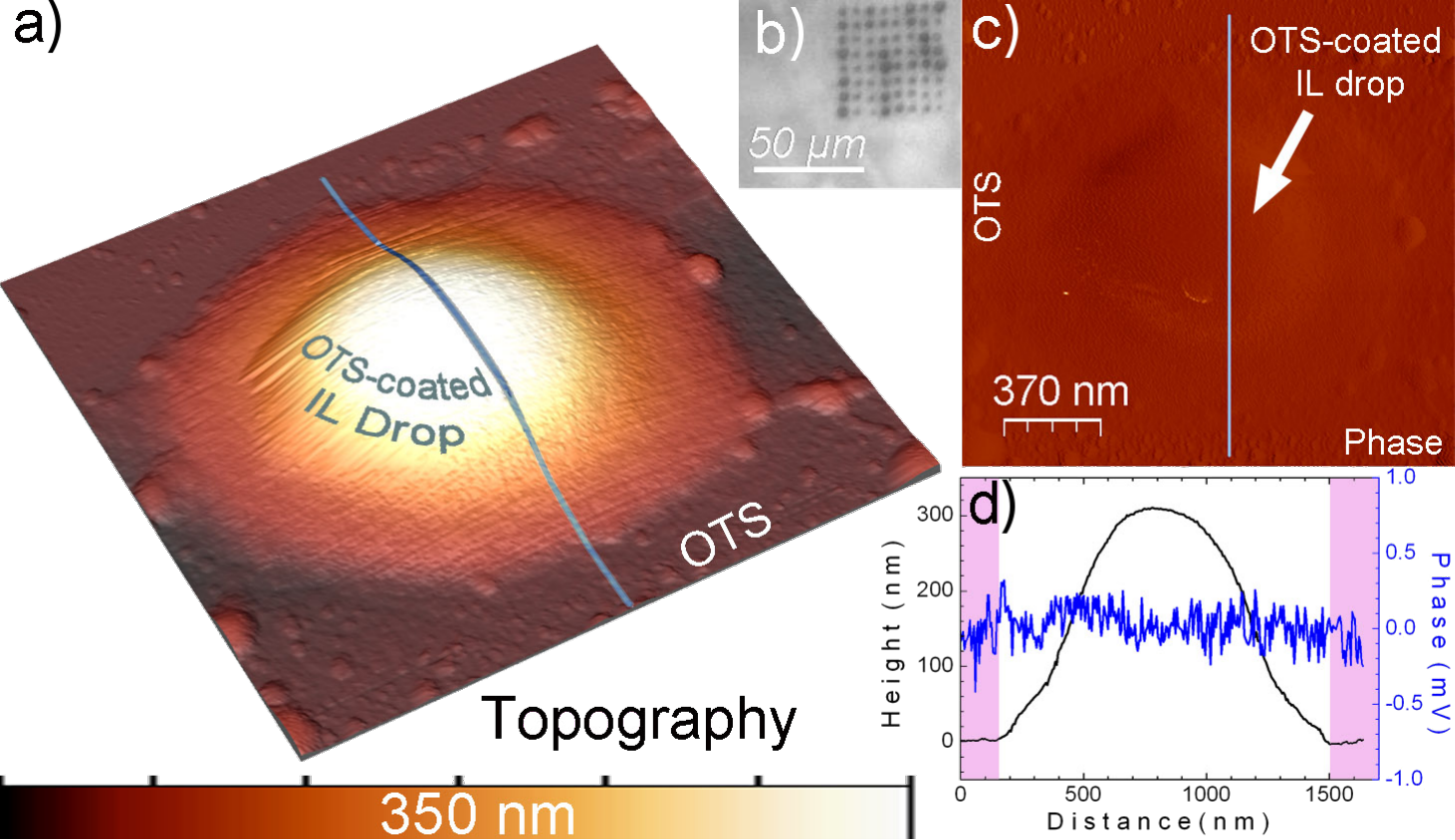
a) A representative OTS-coated [Bmim]Cl drop on the OTSpd pattern. AC mode topography image. b) Optical image of OTS-coated [Bmim]Cl drops on the OTSpd disc array. The imaged was acquired under water. c) The phase image corresponding to the topography image in a). d) The topography (black line) and phase (blue line) channel cross-sectional profiles corresponding to the cyan lines shown in a) and c). The topography cross-section profile reveals that the drop height is 300 nm. The phase cross-section profile indicates that the phase signal of the OTS regions (pink zones in d) and the phase signal of the OTS-coated drop surface are the same because their difference in phase signal is smaller than the noise level.

Silane molecules react with water to form silanols, which subsequently cross-link with each other using the Si–O–Si covalent bonds and form a polymer network [15–16]. Such a silane network is mechanically stable and chemically inert. When silane molecules react with hydrophilic surfaces, a self-assembled silane layer is formed on the surface. The cross-linked silane film can be formed on the IL drop surface as well because water adsorbed there. In our experiment, we incubated IL drop arrays in OTS vapor. We found that an OTS layer covered the IL drop surface, forming a capsule that encapsulated the IL inside (Figure 2). The capsule had the same Mexican hat shape of the IL assembled on the OTSpd disc. The hemispherical cap shaped drop is in the center on the OTSpd disc and co-exists with the surrounding precursor layer (the rim of the Mexican hat), which covers the remaining OTSpd disc. The height cross-sectional profile along the cyan line in the AC mode topography image (Figure 2a) is plotted in Figure 2d (black line), which reveals that the drop in Figure 2a is 300 nm in height. Figure 2b is an optical image of the OTS-coated [Bmim]Cl drop array. The image was acquired under water after 1 h of incubation. Under the optical microscope, interfaces separating the drop and the water can be clearly observed. In contrast, in the control experiment for those IL drops assembled on OTSpd disc without OTS coating after water was applied to the patterned area, the un-coated IL drop was instantaneously miscible with water, and thereby disappeared.

Because the coated-drops still existed after incubation, we conclude that the coating was a complete layer which can separate [Bmim]Cl inside the capsule and the water outside the capsule. In the phase image (Figure 2c) corresponding to the topography image shown in Figure 2a, the phase signals over the drop and the OTS background are the same. This can be further quantitatively illustrated by the phase cross-sectional profile along the cyan line in Figure 2c which is plotted as the blue line in Figure 2d. In the plot, the phase signal difference between the drop surface (central white region in Figure 2d) and the OTS film (pink regions in Figure 2d) is smaller than the noise level. Thus, we conclude that OTS and the drop surface have the same phase signal. The phase signal acquired during the same scanning line and under the same instrumental set-ups represents the surface identity. Since the OTS background is methyl-terminated, we conclude that the vapor treated [Bmim]Cl drop is also covered with a layer of methyl-terminated OTS silane.

During the AC mode imaging, we also varied the tapping amplitude set point in order to examine how the encapsulated IL responds to different external tapping intensities. At a high amplitude set point (99.5% of the free oscillation amplitude), the tip tapped the OTS-coated drop gently. A smooth topography profile of the drop was acquired. In contrast, at a low set point (95% of the free oscillation amplitude), the tip tapped the OTS-coated drop hard, with a high force. Phase signal oscillations were observed when the tip scanned over the drop, as shown in the Supporting Information File 1, Figure S1. The oscillation at a low set point indicates that the drop was disturbed when the tip tapped it hard which caused the IL inside to oscillate. Hence, the IL inside the capsule was still fluidic. In comparison, under the same low set point, the AFM scan lines over the OTS film background did not show any oscillation because the OTS film was in solid phase. Hence, this control reveals that the oscillation we observed over the drop is the true physical oscillation of the IL inside the drop rather than the electronic oscillation originated from the AFM feedback loop. Therefore, from this experiment we conclude that the coated silane layer only formed at the surface of the IL drop.

### Reaction of the OTS-coated IL capsules

Pinholes widely exist in the silane film that was prepared without stabilization [17]. When the “unstablized” OTS film was imaged using a MikroMasch ultra-sharp AFM tip (~1 nm in tip diameter), no pinholes could be resolved. On the other hand, when the unstablized OTS film is incubated in 11-mercaptoundecyltrimethoxysilane toluene solution, the 11-mercaptoundecyltrimethoxysilane molecules can fill the pinholes in the OTS film, leaving the terminal –SH groups on top. The –SH group can subsequently bond to gold nanoparticles and immobilize them on the surface. Hence, we infer that the size of the pinhole would be around 0.5–1 nm. These pinholes provide spaces for reactions and encounters between the materials encapsulated inside the drop and reactants in the external solvent.

**Figure 3:**
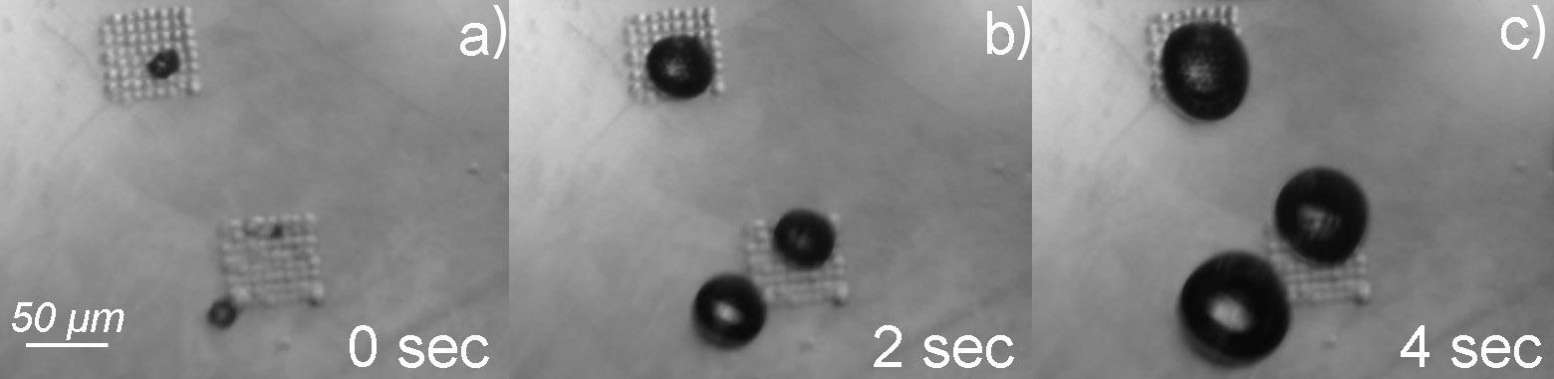
H_2_O_2_ decomposition reaction catalyzed by FeCl_3_. The process was recorded by the optical microscope under 30% H_2_O_2_ solution. The FeCl_3_ [Bmim]Cl solution assembled on two 8 × 8 OTSpd disc arrays. The IL drops were protected by a layer of OTS film coating. a), b), c): The same region with two OTS-coated IL arrays were immersed under 30% H_2_O_2_ solution and recorded at 0, 2, and 4 seconds, respectively. The observed oxygen bubbles (black spots) grew with time, indicating that the H_2_O_2_ decomposition reaction was proceeding.

Figure 3 shows OTS-coated [Bmim]Cl drop arrays. The IL inside drops contained 30% (w/w) of FeCl_3_. The sample was incubated under 30% H_2_O_2_ solution at 25 °C. FeCl_3_ is a homogenous catalyst for the decomposition reaction of H_2_O_2_ [18]. When FeCl_3_ was added in H_2_O_2_ solution, the H_2_O_2_ decomposed and oxygen bubbles were generated in the solution as the decomposition product. In our experimental set-up, the FeCl_3_ was dissolved in IL solution, which was encapsulated by the OTS coating and existed as the immobilized capsules arrays on the designated places on the sample surface. We applied one drop (30 μL) 30% H_2_O_2_ solution onto the surface to cover the OTS-coated FeCl_3_/IL arrays. The Fe^3+^ inside the IL was slowly released from the pinholes on the OTS film. The released Fe^3+^ catalyzed the decomposition reaction of H_2_O_2_, which generated O_2_ bubbles. The reaction was monitored by the optical microscope in real time. As Figure 3 shows, after immersion, oxygen bubbles were observed on the patterned area immediately (within 0.5 s after the H_2_O_2_ drop was applied onto the sample). Under the optical microscope, the smallest bubble that can be resolved is around 600 nm in size, which is at the resolution limit of our microscope. At the beginning, these small bubbles randomly appeared at the surface of the IL capsules. However, nearby oxygen bubbles fused together to form large bubbles. The size of the bubbles increased with time. Upon further growth, the fused bubbles took off from the surface and the patterned area became clean. Then, new bubbles appeared at the interfaces of the OTS-coated IL drops. These new bubbles may not always originate from exactly the same spot in the array as the previous bubbles did. However, the bubbles always started from the OTS-coated IL drops in the arrays. The overall decomposition reaction lasted for ~12 h, until all H_2_O_2_ was consumed. During the reaction, all oxygen bubbles generated from the decomposition were observed to originate from the surface of the IL capsules. This fact suggests that the majority of Fe^3+^ ions did not diffuse into the solution.

The potential leaking of IL into solution was assessed through the following experiment: As the first step, we fabricated one 8 × 8 OTSpd disc array on a 1 × 1 cm^2^ OTS sample. In total, we fabricated three such samples (sample A, sample B and sample C). Within the array, each OTSpd disc has a diameter (*D*) of 3.5 μm. For sample A, we coated the array with IL solution and then coated a layer of OTS silane to encapsulate it. Then, each capsule’s volume can be obtained through the flooding analysis using the AFM topography image. On average, the volume for each capsule was about 8 μm^3^. Then, we put one drop (10 μL) of deionized water over the 8 × 8 OTS-coated IL capsule array for 2 h. Next, we transferred the drop to sample B and let this drop cover on the 8 × 8 OTSpd disc array on sample B. This drop evaporated in air within 30 min. If the IL leaked out from the capsule during the previous 2 h long incubation over sample A, the dissolved IL would be transferred to sample B. Since the IL would not evaporate with the water, IL would be deposited on sample B. Furthermore, because this IL ([Bmim]Cl) does not wet the OTS film, as demonstrated in Figure 1c, the deposited IL would be selectively concentrated on the high-energy 8 × 8 OTSpd disc array on sample B. Therefore, we can use AFM to characterize the 8 × 8 OTSpd disc array to reveal how much IL was deposited. Figure S2 in Supporting Information File 1 shows a representative image of one OTSpd disc after the drop evaporated over the OTSpd disc array. From the image (Figure S2), we computed that the volume of the IL deposition on this OTSpd disc was 0.044 μm^3^_._ We used AFM to characterize all 64 OTSpd discs in the array and computed the IL deposition volume, which yielded an average IL deposition volume of 0.04 μm^3^/disc. AFM scan also revealed that no IL was deposited on the OTS surface. In the control experiment, we put one drop (10 μL) of deionized water over the 8 × 8 OTSpd array for 2 h over sample C, which just had a clean 8 × 8 OTSpd array. After the drop evaporated in air, we characterized the 8 × 8 OTSpd array on sample C. No deposit was found, the OTSpd arrays did not change. Hence, we conclude that the material deposited on OTSpd disc on sample B is the leaked IL. After a 2 h long incubation, only 0.5% (v/v) IL inside OTS-coated capsule was slowly released to water. The OTS coating leads to the slow release of the IL.

The potential leaking of Fe^3+^ from the OTS-coated IL capsule was also studied. We put one drop (30 μL) 30% H_2_O_2_ solution onto the surface to cover the OTS-coated FeCl_3_/IL arrays to initiate the reaction. After 1 h, while the reaction was still proceeding, we used a pipette to transfer the solution onto another clean OTS-coated wafer surface. At this stage, if a large amount of Fe^3+^ was released into the bulk solution phase, the Fe^3+^ would have been transferred onto the clean OTS-coated wafer surface as well. Then, we injected additional 30 μL 30% H_2_O_2_ solution into this drop. Since Fe^3+^ is the catalyst in the decomposition reaction, it will not be consumed. On the contrary, it would continue to catalyze the decomposition reaction. Nevertheless, we did not observe any oxygen bubbles generated within this drop. This fact suggests that the concentration of Fe^3+^ within this 60 μL drop was just too low. The Fe^3+^ concentration in the original 30 μL drop was just twice as high as that of in the 60 μL drop. Therefore, the Fe^3+^ concentration in the original 30 μL drop would be low as well. Our data show that the OTS coating on the IL drop surface effectively suppressed the diffusion of Fe^3+^ into the external solution.

In a separated control experiment, OTS-coated FeCl_3_-free IL drops were incubated with H_2_O_2_ solution. No oxygen bubbles were generated, indicating that Fe^3+^ was responsible for the decomposition of H_2_O_2_. From these experimental results we conclude that the H_2_O_2_ decomposition reaction occurred at the IL–OTS–water interface. The reaction occurred either because the Fe^3+^ ions diffused out of or the H_2_O_2_ molecules diffused into the IL capsules through pinholes in the OTS film.

## Conclusion

We found that lyophilic carboxylic acid-terminated OTSpd chemical pattern can direct the assembly of the IL on the OTS film surface. The chemical pattern can control the position, size and shape of the IL on the surface. The IL drops assembled on the chemical patterns can be coated with a protective layer of silane which encapsulates the IL and the solute within the IL. The coated IL drops can stably exist in other solvents that are miscible with the IL. Pinholes in the silane coating layer enable a slow material exchange between both sides of the protective silane layer.

Our experiments show that the FeCl_3_ catalyst encapsulated within the IL drop can still catalyze the decomposition reaction of the hydrogen peroxide at the IL–OTS coating–water interface when the coated IL drops were immersed in hydrogen peroxide solution. Therefore, the coated IL drop may allow homogenous catalytic reactions to proceed in a heterogeneous fashion at the designated places. This capability provides conveniences for the subsequent product separation procedures.

## Experimental

### Instruments

The chemical pattern fabrication and characterization were conducted by the Agilent PicoPlus 2500 environmental AFM. The optical examination of the surface was conducted using a Nikon Eclipse 55c microscope.

### Procedures

The silicon wafers (Nitrogen doped, resistivity 1–40 Ω∙cm) were polished to an ultra-flat level (root mean square roughness <5 Å) and were then cut into 1 × 1 cm^2^ pieces. The wafer samples were cleaned by piranha solution (1 part of 98% H_2_SO_4_ and 2 parts of 30% hydrogen peroxide) at 125 °C for 15 min. After rinsing the samples in deionized water and drying in an ultrapure nitrogen environment, the cleaned samples were immersed in a 5 mM OTS (octadecyltrichlorosilane, 97%, Gelest, Inc) toluene solution for 12 h at 20 °C in order to form an OTS film on the sample surface.

Next, the OTS-coated samples were rinsed in toluene and annealed in a sealed vial at 40 °C, 100% relative humidity (RH) for 12 h. Subsequently, the samples were incubated in a 5 mM OTS toluene solution again. The stabilization (*rinsing-annealing-OTS solution incubating*) process was repeated for three times in order to remove the pinholes inside the OTS film [17,19–21]. The final OTS film was an ultra-flat, pinhole-free, featureless film.

The OTSpd patterns were fabricated by the scanning probe deep oxidation lithography. In a 100% RH environment (at 25 °C), a Pt–Ti coated conducting AFM tip (CSC-17 Pt–Ti, from MikroMasch) was used to contact the OTS-coated sample. A 10 V voltage was applied to the silicon wafer, whereas the conducting AFM tip served as the ground. Due to the bias voltage, OTS under the tip was oxidized and degraded into carboxylic acid-terminated OTSpd pattern. Several 8 × 8 OTSpd disc arrays were fabricated on the OTS film. The size of the array was 50 × 50 μm^2^.

### Coating IL on OTSpd patterns

[Bmim]Cl was purchased from Sigma-Aldrich. It has a melting point of 70 °C and an advancing contact angle of 88° on OTS film [22]. In a sealed vial, 10 g [Bmim]Cl powder was heated to 120 °C and then cooled to room temperature. After cooling, [Bmim]Cl in the vial existed as a viscous super-cooled liquid at 25 °C. A drop of [Bmim]Cl was placed on the patterned area on the OTS-coated sample. Then, we used a pipette to remove the IL drop from the sample surface. After contacting the patterned surface, the IL assembled on the OTSpd patterns as drops.

Samples with IL drops assembled were placed in a sealed vial with 33 μL OTS. The vial was incubated at 55 °C for 2 h. The OTS molecules from the vapor formed a layer at the IL drop surface.

## Supporting Information

File 1 Supporting material.
Figure S1: The oscillation during the AC mode scanning of an OTS-coated IL drop.
Figure S2: Assessment of IL leaking from the OTS-coated capsule.
