# Supplementary material for: Octadecyltrichlorosilane (OTS)-coated ionic liquid drops: Micro-reactors for homogenous catalytic reactions at designated interfaces
Source: Beilstein J Nanotechnol. 2012 Jan 12;3:33–9. doi: 10.3762/bjnano.3.4 (PMC3304328; doi:10.3762/bjnano.3.4)
Supplement: File 1 — Supporting material. Figure S1: The oscillation during the AC mode scanning of an OTS-coated IL drop. Figure S2: Assessment of IL leaking from the OTS-coated capsule. [file Beilstein_J_Nanotechnol-03-33-s001.pdf]

Supporting Information

for

Octadecyltrichlorosilane (OTS)-coated ionic liquid drops: Micro-reactors for homogenous catalytic reactions at designated interfaces

Xiaoning Zhang and Yuguang Cai*

Address: Department of Chemistry, University of Kentucky, 505 Rose Street, Lexington, Kentucky, 40506, USA

Email: Yuguang Cai - ycai3@uky.edu

* Corresponding author

Supporting material

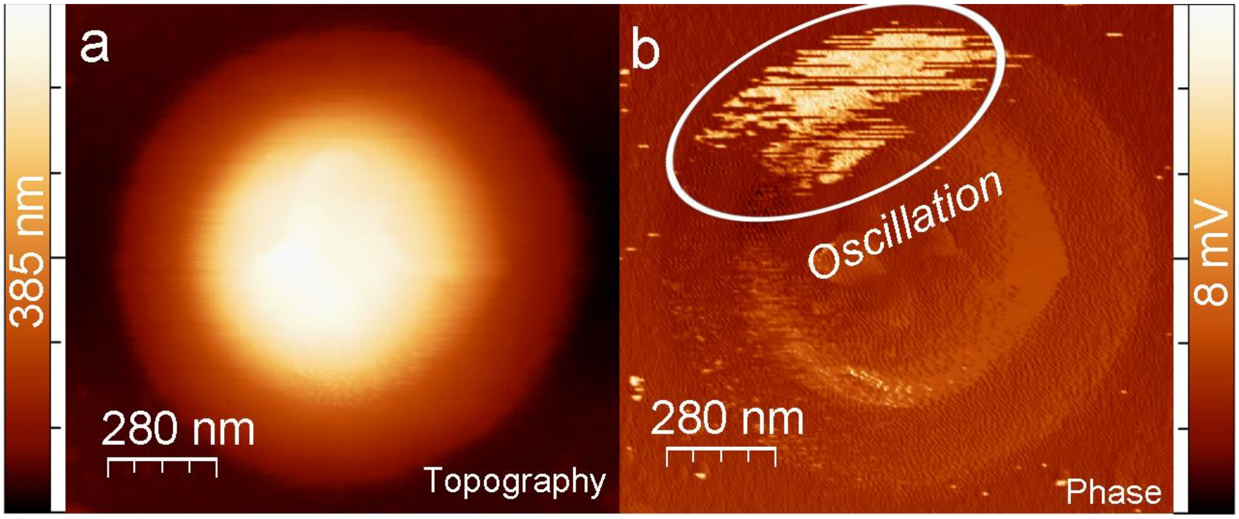

Figure S1: AC mode image of an representative OTS-coated [Bmim]Cl drop. During imaging, the imaging amplitude set point was intentionally varied. When we scanned the upper part of the image b), the amplitude set point was lowered, which led to the tip tap harder on the OTS-coated drop and interacted strongly with the OTS-coated IL drop. As a result, oscillations were observed as highlighted in the white circle in b) Once the set point was increased, the tip tapped the surface gently; the oscillation disappeared, indicating the tip did not disturb the OTS-coated IL drop.

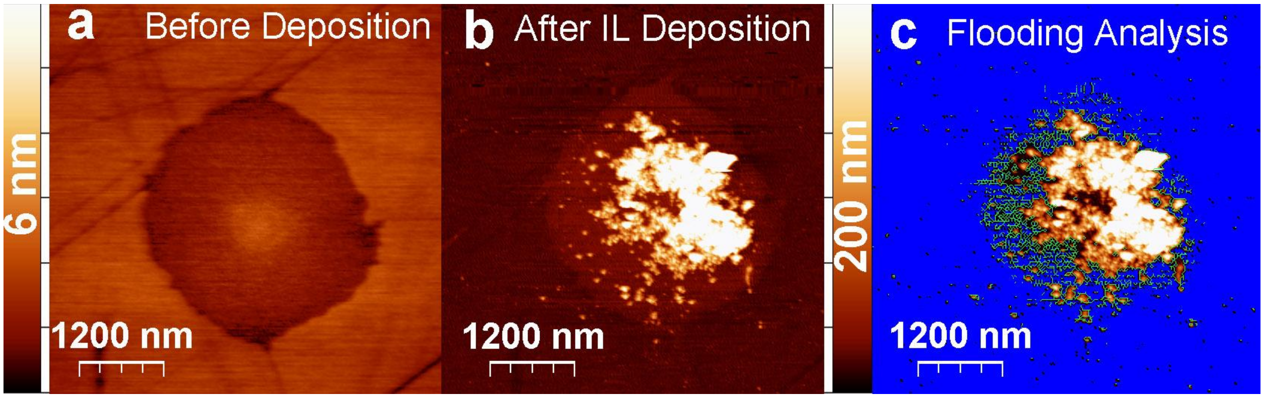

Figure S2: Assessment of IL leaking from the OTS-coated capsule. a) A representative clean OTSpd disc. After a 10 μL drop of water covering the OTS-coated IL capsule for two hours, this drop was transferred onto the clean OTSpd array surface (a) and let this drop to evaporate. b) After evaporation, the IL dissolve in water was deposited on this OTSpd disc. c) Flooding analysis reveals that the volume of the deposited IL is 0.04 μm^3. The green line marks the positions of the IL deposits. The blue region is the flooded area.
